# Remarks on the History and Treatment of Delirium Tremens

**Published:** 1832-03

**Authors:** John Ware

**Affiliations:** Fellow of the Massachusetts Medical Society.


### DELIRIUM TREMENS.

*Remarks on the History and Treatment of Delirium Tremens.* By John Ware, m.d., Fellow of the Massachusetts Medical Society.

(Continued from page 109.)

IV. So far we have referred chiefly to those cases in which delirium tremens assumes the form of a regular paroxysm, terminating in sleep. In acute diseases this is the usual course; but sometimes in the acute diseases, and frequently in the chronic diseases of drunkards, a delirium comes on resembling that of which we have been speaking in everything except that it does not go through the same regular course, or come to a similar termination. Thus, in a case of very severe pleurisy, for two nights in succession, at the height of the disease, the patient was delirious, and resembled so exactly in his appearance, manner, and disordered imaginations, those which are exhibited in delirium tremens, that no one, not acquainted with the whole history of the case, would have suspected that it was anything but a regular case of that disease. The course of the pleuritic affection was not at all modified: the patient was nearly rational through the corresponding days; the delirium subsided rather gradually; he slept occasionally while it continued, and finally recovered, without a proper paroxysm.

In another case of thoracic disease, viz. of inflammation of the lungs, a delirium of the same character came on towards the close of the case, which terminated fatally, continuing for about twenty-four hours, and ending only at death; death taking place as it would have done from the pulmonic affection. I have witnessed other cases of a similar character, in which the delirium resembled that of the regular paroxysm with great exactness, but in which the course that it took, and the mode of its termination, were different.

In chronic diseases, delirium may affect the patient in a similar manner; sometimes occurring every night for a succession of nights, and sometimes only a single night at once, and so returning occasionally for a considerable time. In these cases it would seem to take the place of that delirium which might attend the same diseases in patients not intemperate.

There are also states of disease among drunkards of an anomalous character, affecting at once the mind and body, and approaching very nearly in their aspect, at particular times, to delirium tremens: still they are to be distinguished by the want of regularity in their whole course, by their not constituting a proper paroxysm, and by their having no definite termination in sleep.

Having thus described the general course of this affection, the circumstances under which it originates, and the states of the system and of disease with which it is connected, it may be useful to give a more particular history of its principal symptoms; our previous object having chiefly been to describe their connexion together as constituting the paroxysm. The most remarkable and constant symptoms are the delirium, watchfulness, and tremor.

Of these, the delirium is the most universally and constantly present. It is perfectly peculiar in its character, and so slightly resembles that which is exhibited under any other circumstances, that, if witnessed but for a few moments, one may feel sure with regard to its nature and origin. Its general character is the same throughout the whole of the paroxysm, but the subjects in regard to which it is exercised are as various as the occupations, habits, modes of life, associations, and relations, of the individual attacked.

His imaginary perceptions are generally removed entirely from the actual state of things about him: they often relate to his particular occupation or business, or to whatever other subject may happen at the time to weigh most heavily on his mind. Almost always he imagines himself to be in a different place from that in which he is, and under some disagreeable circumstances. The seaman thinks himself at sea in a gale of wind, and vainly endeavouring to bring his vessel to a safe and proper bearing; the smith at his anvil, labouring ineffectually over a piece of work which he can never finish; the cooper toiling in vain over hoops and staves, which he cannot match; and the ropemaker twisting for ever an interminable length of yarn: all are engaged in a Sysiphian labour, which they are doomed never to accomplish.

But, although the predominating idea for the time has full possession of the mind, and everything is made to conform to it, yet it is frequently changed in the course of the disease, and has sometimes no relation whatever to any of the habits of the patient, or to any circumstances or things with which he is connected. Thus, a patient who had been dissolving a copartnership before his sickness was, in the first place, constantly busied in an entangling controversy about the settlement with his partner; then he suddenly conceived himself to be chased by an alligator, who had been concealed in the chimney of his room; then he would seize upon his bed, and shake it upon the floor, in search of rats and mice, which he supposed to be concealed there, or busy himself in picking lice from his clothes, fleas from his pillow, or hairs out of his drink.

There is in the aspect and conduct of those affected by this delirium a very peculiar and strong impression of reality: this is sometimes exhibited in a manner sufficiently ludicrous, as in the case of a cooper, who insisted that his mother, a woman of a round and plump figure, was a hogshead which he was to hoop. At other times the spectacle is painful: nothing can be more real than the expression of horror, fear, or despair, which are occasionally witnessed in the unfortunate subjects of this disease. The dread of robbery and of murder are as distinctly produced in their minds as they can be in those of persons actually subjected to these dangers. There is often a thrilling and almost startling truth in their expressions of voice and countenance; and, from the entire absence of any of the proper exciting causes of such emotions, the whole scene appears to the bystander like excellent acting.

The presence of a stranger, and more particularly of the medical attendant, is almost always sufficient to calm, for a short time, the most violent of these patients, and even to suspend the current of their imaginations: it is only, however, for a short time; for, if the visit of the physician even be prolonged to any considerable length, his authority is lost, and the delirium returns in its full violence. I once sat beside a patient for an hour or two in the beginning of the evening, when the paroxysm was coming on, with the hope of being able to keep up that kind of influence which I found was at first exerted over him. He was a person of character and education. For some time, by speaking decidedly to him when attempting to rise from his bed, at the same time lifting up my finger as if to indicate the importance of silence and quietness, I succeeded in inducing him to throw himself back and remain still, though looking wildly around, and talking incoherently of things which he supposed to be going on about him. Suddenly he started up, escaped from the opposite side of the bed, and immediately attempted to jump from a window that was near. After his recovery, he for some time believed that I had sat by him with a pistol in my hand, which I pointed at him whenever he attempted to get up or to escape: the impression thus produced on his mind was very disagreeable, and was not obliterated for a considerable time.

Patients labouring under delirium tremens are not disposed to commit violence or do mischief intentionally; and, although it is very common for them to tear their clothes and break furniture in pieces, yet it is generally with the intention of bringing about some important purpose, which they imagine they can thus accomplish. There is nothing morose or sullen in the temper they display; indeed, they are usually timid, irresolute, and easily alarmed. The apprehension of some design upon them is often the predominating feeling in their minds, and they as frequently imagine that they have already suffered some severe injury. They are in fear of sheriffs, of robbers, of being murdered, &c.; they commonly believe that they have been carried away, and are forcibly detained from home; they often start at any loud and sudden noise, thinking that a musket has been fired at them. One patient declared that he had been flayed, and as a proof pointed to the bare flesh of his arm, from which, as he said, the skin had been taken; another asserted that he had been taken to pieces, and put together again. In the state of extreme terror to which these various apprehensions reduce them, it is not uncommon for them to attempt jumping from windows, and this they sometimes accomplish.

I know of but one individual who has committed any violence on himself: he did this in two several attacks. In the first he had suffered very severely from pain in the head, was much dejected, and impressed with some undefined expectation of evil: he mangled his throat with a penknife, bled profusely, but was prevented from further mischief, and his paroxysm went through its usual course. In the second attack, he made a similar attempt with a razor, wounded some small arteries, and cut badly into the larynx: he bled to faintness, and was much reduced by the hemorrhage, but his disorder was not affected by the loss of blood, and he finally recovered.

There is hardly anything in disease more remarkable than the spectacle exhibited by a patient in the height of a paroxysm of delirium tremens. We see him intently engaged in the pursuit of some imaginary object, labouring with the utmost diligence and earnestness upon imaginary materials, and with imaginary companions; his countenance haggard, and worn by anxiety and watchfulness; and his hair, face, and limbs bathed in a profuse sweat. At one time we find him supporting with his whole strength the wall of the house, believing that it is about to fall in and crush him; at another time he is engaged in a combat with snakes, alligators, rats, mice, or insects, of which his room, his bed, and his clothes are full; at another, his flesh is filled with pins, needles, fish-hooks, or pieces of glass, of which he is endeavouring to get free, cutting himself even to the quick in the attempt; at another, he is in an agony of terror, trembling in every limb at the fear of murder or fire, and beseeching in the most piteous accents for assistance; again, perhaps, we may visit him when he is tranquil and comparatively calm, and ready to entertain us with a long and solemn narrative of the dangers and adventures of the night before.

But, strong as must be the impressions upon the mind which are thus exhibited, they are very evanescent, and, with few exceptions, continue for but a short time after recovery. It seems to the recollection of the patient as if the imaginations of his diseased state were the occurrences of a troubled dream; there is the same kind of mistiness and uncertainty about the former that there is about the latter: in short, the state of the mind in delirium tremens very closely resembles that which exists in dreaming, whilst the state of the body differs. Not only is the imagination of the patient filled with the objects which form the subjects of his delirium, but the perceptive powers partake in the same unnatural state: with his senses open to external impressions, he sees, hears, and speaks to and of persons and things, which in his sleep he sees, hears, and speaks to, only in imagination. There is in each case the same want of the corrective power of the judgment; the mind follows on passively in the train of its associations, without any attempt to correct their incongruity.

In corroboration of this view, it may be remarked that the delirium often seems to be merely a waking continuation of that state of mind which has existed during sleep for several preceding days. Before the paroxysm begins, the patient describes to you his dreams as being of precisely the same character that his waking imaginations afterwards are when the disease has become established: in fact, it is occasionally difficult, about the time of the access of the paroxysm, to determine whether he has slept or not, so blended are the states of mind in sleeping and waking, and so insensibly does he pass from the disturbed sleep which precedes the disease to the disease itself.

The correcting power of the judgment is not always entirely lost in sleep: we are sometimes able to reason concerning the probability of our fancies. This happens occasionally in delirium tremens: a person of very strong mind may sometimes detect the fallacy of his imaginations, and obtain a partial control over them; this lasts but for a moment. I once succeeded in convincing a patient who thought himself away from home in a strange place, that he really was in his own house, by directing his attention strongly to several pictures which were hanging around his room, and to the peculiarities in its arrangement, furniture, &c. He was convinced for the moment that he *was* at home, but not that he *had been* at home: he wondered how he had got back so quick. He soon relapsed into his original state.

We often find that events happening about us, which strongly appeal to the senses, become parts of our actual dreams, by means of certain quick operations of our minds by which they are suddenly incorporated with them: thus, the shutting of a door, the speaking of a few words, the striking of a bell, whilst we are asleep, often become part of our dreams. It is so in delirium tremens: things actually happening enter into and become part of the waking reverie, after being magnified or exalted by the excited imagination; the shutting of a door is taken for the report of muskets, the *singing* of wood on the fire for the music of a band, &c.

There is a resemblance also between the state of mind in delirium tremens, and that which exists in the affection which has been denominated Ecstasis, or ecstacy, of which examples are not unfrequent. The resemblance, however, extends no further than to the general laws according to which the mind is affected: in other respects there is none.

Next to the delirium, the watchfulness is the most remarkable symptom: so characteristic is it, that it has been proposed, as affording a better distinguishing appellation of the disease than the tremor; and it is unquestionably true that the tremor is more frequently absent than the watchfulness, and that Delirium vigilans* is a more expressive name than Delirium tremens. Uniformity is, however, so much more important than significance of nomenclature, that it seems not desirable to attempt to substitute a new name for that now in general use.

The termination of a paroxysm of delirium tremens is always, as has been already mentioned, by a profound sleep, and no cessation of the delirium or other symptoms is to be regarded as indicating a favorable close of the disease, unless it has been preceded by it. Sleep, however, is not always to be regarded as indicating the speedy termination of the paroxysm, since it is not uncommon for patients to sleep a little (from a few minutes to an hour, for instance,) on each day of the delirium; and this is more likely to happen when the attack takes place in the course of some other disease. Lucid intervals are not common, but they sometimes occur, and so far as a few cases can go towards establishing a general principle, we are to regard them, when occurring before the regular termination of the paroxysm, as unfavorable indications. Two cases only, however, of this kind have fallen under my observation, and I do not recollect that any others have been recorded.

In the first, the disease began with convulsions, which were repeated during the first twelve hours: on the second morning, without having slept at all, the patient had a perfectly rational interval, of considerable duration, and talked with his friends and attendants in a manner which would have led no one to suspect him of having laboured under any alienation of mind. In a few hours, however, the delirium returned, and he died in about forty-eight hours from the first attack of convulsions.

In the second case, the disease, though finally assuming all the peculiar symptoms of delirium tremens, and occurring in a person who had once suffered from it, did not exhibit itself in an unmixed form: his symptoms were at first irregular, and arose (as I conjectured,) from a combination with

* Dr. Hayward, of Boston, in the New-England Medical Journal, vol. xi., for 1822, advocates the substitution of this name for that now in use, by arguments for its adoption, which would be unanswerable were the disease to be now named for the first time.

inflammation of the brain, but they assumed finally the aspect of genuine delirium tremens. After one or two days of delirium, and a night passed without sleep, he became, for nearly a whole day, perfectly rational. This relief followed venesection and the free operation of cathartic medicines, but was not preceded by sleep, and was not relied on as affording a favorable prognosis: the symptoms accordingly returned with increased violence, and with a more close resemblance to delirium tremens. Sleep was procured by laudanum, but without any relief of the other symptoms, and the case terminated fatally.

The tremor which has given its name to this disease is nearly as universal a symptom as the watchfulness; but, though present during some part of almost every case, is not uniformly present throughout its whole course. It is occasionally very violent, and reminds one of the shaking of the limbs in an attack of intermittent; but it sometimes amounts merely to a slight tremulousness. It extends to most of the voluntary muscles, affecting the tongue, the lips, the eyes, the limbs, and the muscles of respiration; the affection of the latter being indicated by the tremulousness of the voice, and of the sound produced by the air in inspiration.

We may make this additional distinction between the watchfulness and tremor, as serving to characterise the disease, that the former occurs only in this affection, whilst the latter makes its appearance in all cases of sickness among drunkards, and is even common in many who are in their usual health. No doubt the existence of the tremor in a case of sickness should lead us to suspect the approach of delirium tremens, but it affords no certain indication.

Besides these, which are the most characteristic symptoms, others occur, of more or less importance.

Convulsions are not unfrequent: they are often the first symptom which excites notice; they are sometimes the immediate precursors of death at the close of the paroxysm; and they occasionally take place in its course, and sometimes bring it prematurely to a fatal termination; they are always an unfavorable, but by no means a fatal symptom, though they perhaps appear in a majority of cases which end in death. We may reasonably regard them, when they begin the disease, as implying some primary affection of the brain. In a few cases which I have had an opportunity of examining after death, where convulsions had preceded, effusion has been found to have taken place both on the external surface of the brain and within the ventricles.

Patients are seldom without some unnatural sensation in the head: this, in many cases, amounts to a severe headach, and may be the first symptom of which complaint is made; sometimes it is only a dizziness, heaviness, or sense of confusion. If the patient, even in the height of the delirium, be asked how he is, he perhaps answers abruptly that he is "pretty well," "quite smart," but that he "feels badly about the head;" and he seldom fails to acknowledge some feeling of this kind, at whatever period of the case inquiry is made.

In one instance the patient complained that surrounding objects appeared to him to move to and fro; but he was aware that this arose from a morbid affection of the sense of vision, and did not confound it with the delirious ideas which occupied his mind.

The pulse varies much in frequency in different cases, and at different periods of the same case: at first it is often of the natural standard, becoming rapid as the case proceeds; sometimes it is frequent from the first; almost always in the height of the paroxysm, it becomes very rapid, rising to 130, 140, or even 150: still in a few cases it continues at the natural standard, or a little above it, to the termination of the disease in sleep; and such cases rarely do otherwise than well. If the pulse do not rise above 100, we may regard the case as almost certain to do well, and the danger increases as the pulse rises: still even a quick pulse is not a very fatal symptom, since persons often recover whose pulse has risen to 130 and 140; and a slow pulse is not unequivocally favorable, for I have known a patient carried off very suddenly by convulsions, whose pulse had not exceeded ninety. When the pulse, after having been very quick and small, becomes slower and fuller, particularly when this happens towards the close of the paroxysm, we may predict a favorable event with considerable confidence. This change in the pulse often precedes, by a few hours, the termination of the disease in sleep. When, on the contrary, at the proper time for the conclusion of the paroxysm, the pulse becomes quicker, smaller, and weaker, there is reason to fear an unfavorable event, though the indication in this case is less certain than in the former.

There is nothing peculiar in the state of the tongue: it is commonly preternaturally clean, red, and tremulous; but this appearance is common in the diseases of drunkards. It is sometimes covered with a thin white fur, more rarely with a thick; it is very seldom dry, excepting after great and exhausting muscular exertion: sometimes it is protruded, and kept so, with difficulty; and often, at the beginning of the disease, the patient, when asked to shew his tongue, thrusts it out very suddenly, with some distortion of the countenance, and a staring expression of the eyes. In general, we may regard the tongue as rather indicating the general state of the system, than the state of the disease itself.

The appetite usually fails, partially at least: in some cases it has remained good, and the patient has been allowed to take his regular meals. It has always appeared to be a favorable symptom.

Thirst is seldom excessive; less so than in most diseases accompanied with excitement. In that form of it which follows immediately from excess in ardent spirits, the desire for them may remain, and sometimes in cases of a different description; but often there is no such indication.

The skin is generally soft and moist from the first, but, toward the close of the disease, it is bathed in a very profuse sweat. This may be partly attributed to the muscular exertions of the patient, but not entirely, since it continues after he has fallen asleep. Toward the close of the paroxysm, the hands and feet, and often the whole body, become cold, though still covered with sweat, and this more particularly in those cases which have a fatal issue.

The countenance, in the early stages of delirium tremens, has merely a wild and unsettled look: in the advanced periods, particularly in bad cases, it is anxious and troubled, and, during the last few hours before the close of the paroxysm, becomes strikingly haggard and ghastly.

In forming an opinion with regard to the probable event of any case of this disease, we are to be governed by a variety of considerations, the most important of which may be gathered from the preceding remarks. In patients of the first class, or those in which the attack arises immediately from excess, the danger may be regarded as almost nothing. In cases of the second class, the danger is undoubtedly greater, but is still very small, unless there have been several previous attacks. In those of the third class, the danger is always considerable, but the degree of it will depend chiefly on the nature and severity of the original disease. Where the local affection is slight, such as an inconsiderable external injury, or a common catarrh, the risk of death is but little greater than in an uncombined attack; but where it is severe, as in inflammation of the lungs, in dysentery, or in compound fracture, the patient is in great peril. The danger is greater when the delirium comes on at the height of an acute case, than when it occurs after an alleviation or remission of the symptoms: indeed, the most frequently fatal cases are those already referred to, where the disease does not go through the regular course, but simply takes the place of the delirium which comes on at the height of many acute cases, and precedes death but for a short period.

We can seldom be justified in giving an unqualified opinion of the event of a case of delirium tremens, either in favor of or against the recovery of the patient: it is often a matter of much delicacy so to state the possibilities of the case as not, on the one hand, to lead the friends to too sanguine an expectation of recovery, or, on the other, to too unfavorable a view of the result. Degraded as the habits are which lead to this disease, and lost to all that is honourable or desirable in life, as most of the subjects of it are, still some are not so, and many, even of those who are, are objects of affectionate solicitude to parents and friends. Every physician must meet with many cases where the feelings of those around the patient demand the utmost consideration and sympathy, even if his own character claim no respect. It is not uncommon for this disease to occur in young men who are objects of interest to highly respectable families; in husbands who have wives and children dependent on them: and even in wives and mothers themselves. Some of the most painful scenes we can witness are connected with instances of this kind, not only on account of the patients themselves, but of those also who are connected with them, and are interested in their recovery. Indeed, there is hardly any disease, for the recovery of friends from which there is more anxiety manifested, than there sometimes is in this; from the hope, so generally a fallacious one, that the sufferings of sickness and the danger of death may serve to reclaim the patient from the course which has subjected him to them.

On account of the friends, therefore, it is necessary to be careful in the opinion given of the probable event; for there is hardly any case, even those which present the most favorable appearances, which may not terminate fatally, and that very suddenly. The possibility, therefore, that such may be the event should always be stated fairly, and with proper qualification; the favorable indications being allowed their due weight. In this way, the minds of those interested will be in some measure prepared for the worst that may ensue, and yet not unnecessarily disturbed by the anticipation of an inevitable evil. This, whilst it is the course most consistent with exact truth, so far as our knowledge of disease enables us to judge, is also, on the whole, the kindest toward those who are interested; for a few days' qualified apprehension of danger is far better than a feeling of security which is founded on ignorance of the real probabilities of the case, and which leads to so much distress when the event shews it to have been false.

(To be continued.) p. 280.

